# Hepatitis C Virus RNA-Dependent RNA Polymerase Is Regulated by Cysteine S-Glutathionylation

**DOI:** 10.1155/2019/3196140

**Published:** 2019-09-03

**Authors:** Marina K. Kukhanova, Vera L. Tunitskaya, Olga A. Smirnova, Olga A. Khomich, Natalia F. Zakirova, Olga N. Ivanova, Rustam Ziganshin, Birke Bartosch, Sergey N. Kochetkov, Alexander V. Ivanov

**Affiliations:** ^1^Engelhardt Institute of Molecular Biology, Russian Academy of Sciences, Moscow, Russia; ^2^Université Claude Bernard Lyon 1, INSERM 1052, CNRS 5286, Centre Léon Bérard, Centre de Recherche en Cancérologie de Lyon, Lyon 69434, France; ^3^Shemyakin-Ovchinnikov Institute of Bioorganic Chemistry, Russian Academy of Sciences, Moscow, Russia

## Abstract

Hepatitis C virus (HCV) triggers massive production of reactive oxygen species (ROS) and affects expression of genes encoding ROS-scavenging enzymes. Multiple lines of evidence show that levels of ROS production contribute to the development of various virus-associated pathologies. However, investigation of HCV redox biology so far remained in the paradigm of oxidative stress, whereas no attention was given to the identification of redox switches among viral proteins. Here, we report that one of such redox switches is the NS5B protein that exhibits RNA-dependent RNA polymerase (RdRp) activity. Treatment of the recombinant protein with reducing agents significantly increases its enzymatic activity. Moreover, we show that the NS5B protein is subjected to S-glutathionylation that affects cysteine residues 89, 140, 170, 223, 274, 521, and either 279 or 295. Substitution of these cysteines except C89 and C223 with serine residues led to the reduction of the RdRp activity of the recombinant protein in a primer-dependent assay. The recombinant protein with a C279S mutation was almost inactive in vitro and could not be activated with reducing agents. In contrast, cysteine substitutions in the NS5B region in the context of a subgenomic replicon displayed opposite effects: most of the mutations enhanced HCV replication. This difference may be explained by the deleterious effect of oxidation of NS5B cysteine residues in liver cells and by the protective role of S-glutathionylation. Based on these data, redox-sensitive posttranslational modifications of HCV NS5B and other proteins merit a more detailed investigation and analysis of their role(s) in the virus life cycle and associated pathogenesis.

## 1. Introduction

Hepatitis C virus (HCV) is a widespread human pathogen that in 80% cases establishes persistent replication in the liver [1]. The WHO estimates 0.5-2% of the world population to be chronic hepatitis C (CHC) carriers. CHC is often accompanied by persistent inflammation, fibrosis, and various metabolic disorders in the liver [2]. All these pathologies contribute to a possible development of liver cirrhosis and hepatocellular carcinoma. Together with hepatitis B virus (HBV), HCV accounts for approximately 80% of HCC cases in the world [3]. Since 2011, multiple compounds that target enzymatic activity and/or functions of the HCV protease (NS3 protein), RNA-dependent RNA polymerase (RdRp, NS5B protein), and the regulatory NS5A protein were approved for the treatment of CHC [4]. Using treatment combinations, sustained virological response rates have reached 95-99% (for example, [5–7]). At the same time, clearance of the infection does not reduce risks of the development of liver cirrhosis and cancer to the initial level, observed in the uninfected population [8]. Scarce studies provided evidence that even acute hepatitis C that clears spontaneously leads to an elevated rate of liver pathologies. So, the investigation of the interplay between the virus and host cell processes requires additional endeavors.

One of the factors that contributes to liver pathologies is the disturbance of the redox status of the host cell (reviewed in [8]). It results from enhanced production of reactive oxygen species (ROS) and altered expression of ROS-scavenging enzymes. ROS represent a spectrum of short-lived oxygen intermediates including hydroxyl radicals (HO^·^), superoxide anions (O_2_^·-^), hydrogen peroxide (H_2_O_2_), and others [9]. As several types of ROS have a very high oxidizing potential towards various biological molecules, enhanced ROS production above the levels that can be scavenged by the host antioxidant systems was considered hazardous for cells. At the same time, there are mounting pieces of evidence that ROS play signaling and regulatory functions and thus are indispensable for cells [10]. So, in recent years, the redox community shifted away from measuring and identifying ROS towards the identification and investigation of redox switches, *such as oxidation* of cysteine -SH groups into sulfenic (-SOH) or sulfinic (-SO_2_H) acid residues, and S-glutathionylation (*i.e.*, coupling to glutathione via disulfide bond), and to assess the consequent impact on protein structure, stability, and function. Oxidation changes for example the enzymatic activity of protein phosphatases (PTPs) [11] and thus affects signaling pathways, hyperoxidation-driven gain of chaperone activity by peroxiredoxins [12], and redox-driven glutathionylation of Na^+^/K^+^-ATPase that abolishes their activity and thus changes the levels of monovalent ions in cells [13].

However, much less is known about redox biology of viral infections. With few exceptions, studies so far focused on the investigation of mechanisms by which various viruses augment ROS production and solicit expression of antioxidant enzymes such as catalase (CAT), superoxide dismutases (SOD), or glutathione peroxidases (GPx) (as reviewed in [8, 14, 15] and other reviews). In addition, some efforts were given to establishing correlations between levels of ROS production and virus-associated pathologies [8, 14–17]. In contrast, almost no attention was given to a possibility of redox posttranslational modification of viral proteins and their role in virus life cycle and pathogenesis. The only exceptions are the reported S-glutathionylation of retroviral proteases [18–20], nsP2 of chikungunya virus [21] and of NS5 protein of Zika, and dengue [22].

There are many pieces of evidence that show that HCV triggers massive ROS production that is even higher than that observed in HBV infections. HCV induces mitochondrial dysfunction and induces ROS-producing enzymes such as NADPH oxidases (Nox) 1 and 4 [23–26], cytochrome P450 2E1 [23, 24], and ER oxidoreductin 1*α* (Ero1*α*) [23]. HCV also affects the expression of antioxidant enzymes including those regulated by the Nrf2 transcription factor, although the results from different groups remain controversial [27–29]. Several studies also suggest that replication [30] and production of infectious virions [31] are suppressed by lipid peroxides whose production is augmented by the infection. However, a possibility of a direct modulation of HCV protein functions by ROS or other reactive species, as well as redox-sensitive cysteine modifications, remains unexplored. In the presence of the virus or its proteins, production of hydrogen peroxide is augmented, which can lead to oxidation of cysteine residues. In addition, HCV causes elevation of the levels of oxidized glutathione that may drive S-glutathionylation of cellular and viral proteins. So, our goal was to investigate if the NS5B protein is subjected to S-glutathionylation and if such a modification may play a role in regulation of NS5B RNA polymerase activity.

## 2. Experimental Section

### 2.1. Reagents

The plasmid pJFH1-SGR and Huh7.5 cells were courteously provided by Prof. C.M. Rice (The Rockefeller University, New York, USA) and Apath, L.L.C. (St. Louis, MI, USA). Restriction endonucleases were from Fermentas (Vilnius, Lithuania), and oligonucleotides were synthesized by Evrogen JSC (Moscow, Russia). DNA purification kits and DNA polymerases were also provided by Evrogen. Ni-NTA-agarose and Rosetta(DE3) *Escherichia coli* were purchased from Novagen (Madison, WI, USA). Most of other reagents were from Sigma-Aldrich (St. Louis, MO, USA) if not stated otherwise.

### 2.2. Construction of the Plasmids

The plasmid pET21-2c-NS5B*Δ*21 encoding the C-terminally truncated protein of the JFH1 isolate was constructed using the two-cistron vector described previously [32]. The protein-encoding DNA fragment was amplified from the plasmid pJFH1-SGR using the oligonucleotides 5′-AATGAATTCCATGTCATACTCCTGGAC-3′ and 5′-AATCTCGAGGCGGGGTCGGGCGCGC-3′. The resulting DNA fragment was digested with endonucleases *EcoR*I and *Xho*I and cloned into the respective sites of the vector pET-21-2c.

Construction of the plasmids for the expression of NS5B mutants with cysteine-to-serine substitutions was performed according to an approach of Ho et al. [33]. Briefly, the fragments containing the mutation were amplified from the plasmid pET21-2c-NS5B*Δ*21 using two parts each using the oligonucleotides listed in [Supplementary-material supplementary-material-1]. A typical reaction contained 5-10 ng of template DNA, 5 pmol of each flanking primer and mutagenic primer, 200 *μ*M of each dNTP, and 2 units of Tersus polymerase mix (Evrogen) in the recommended buffer. Amplification was obtained after 20 cycles of 15 sec at 95°C, 15 sec at 60°C, and 60 sec at 72°C. Then, 15-30 ng the resulting products representing the “Head” and the “Tail” of the target fragment bearing the mutation were mixed together and subjected to additional 12 rounds of PCR, using 5 pmol of each flanking primer. The products were isolated by agarose gel electrophoresis with subsequent extraction using a Cleanup Standard kit (Evrogen), digested with endonucleases listed in [Supplementary-material supplementary-material-1], and cloned into the plasmid pET21-2c-NS5B*Δ*21 instead of the nonmutated fragment.

Construction of the plasmids encoding HCV subgenomic replicons with substitutions in the NS5B-coding region was performed similarly, using the plasmid pSGR-JFH1 and the oligonucleotides listed in [Supplementary-material supplementary-material-1]. The mutants with substitutions of C89S, C179S, C146S, and C223S were done by amplification of pairs of fragments constituting the region between BsrGI and SnaBI sites, whereas the mutants C274S, C279S, C295S, and D318N, and C521S were constructed by amplification of the SnaBI-EcoRV fragment. In the latter case, the analysis of insert orientation was performed. In case of the C521S mutant, the single SnaBI-EcoRV fragment was amplified, digested with these endonucleases, and cloned as discussed above.

The sequence of all constructs was confirmed by sequencing in Center “Genome” (EIMB, Moscow, Russia).

### 2.3. Expression and Purification of the NS5B *Δ*21 Protein

Wild-type and mutant forms of the NS5B protein were expressed in *E.coli* Rosetta(DE3) strain and purified on the Ni-NTA column as described previously [32] with the exception of absence 2-mercaptoethanol during purification.

### 2.4. RNA Polymerase Activity Assay

The RNA-dependent RNA polymerase activity of the NS5B protein was measured in primer-dependent and primer-independent assays as described previously [32, 34]. When required, the protein was preincubated with 10 mM dithiothreitol, 1 mM GSH, or 1 mM GSSG in the reaction buffer (20 mM Tris-HCl, pH 7.5, 20 mM KCl, 4 mM MgCl_2_) for 10 min in ice prior to the addition of the template or template/primer complex and nucleoside triphosphates to initiate the enzymatic reaction. The reaction was carried out in accordance with the published procedure [32], with the exception of usage of 1 *μ*M UTP.

### 2.5. Western Blotting

The NS5B protein (0.1-2 *μ*g) mixed with a Laemmli loading lacking DTT or 2-mercaptoethanol was incubated at 40°C for 10 min, applied onto 10% SDS-PAGE, and transferred onto a nitrocellulose membrane (GE Healthcare, Little Chalfont, UK) in 25 mM Tris, 192 mM glycine, and 20% methanol, which was blocked with 5% nonfat milk in phosphate-buffered saline (PBS) for 1 h at room temperature (RT). The membrane was probed with a primary antibody to S-glutathionylated proteins (Millipore, MAB5310, 1 : 1000) in PBS supplemented with 0.1% Tween-20 (PBS-T) or with a serum of the rabbit immunized with the recombinant NS5B protein [35] in 5% nonfat milk in PBS-T at 4°C overnight and washed 3 times for 10 min with PBS-T followed by incubation with a secondary antibody: anti-mouse antibody (Santa-Cruz Biotechnology, sc-2005, 1 : 5,000) or anti-rabbit antibody (Santa-Cruz Biotechnology, sc-2005, 1 : 5,000) in PBS-T for 1 h at RT. After three additional washing steps with PBS-T, immunoblots were visualized using an ECL pico detection system (Thermo Fisher Scientific, Rockford, IL, USA).

### 2.6. Mass Spectrometry

The identification of cysteine residues of the NS5B protein that undergo S-glutathionylation was performed by MALDI-TOF MS. The protein was incubated alone or in the presence of 170 *μ*M GSSG for 30 min at room temperature, then with a Laemmli buffer lacking reducing agent for 5 min at 98°C, and loaded onto a 10% SDS-polyacrylamide gel. The band corresponding to NS5B was excised and subjected to in-gel digestion by trypsin [36]. For in-gel digestion, trypsin was dissolved in 50 mM ammonium bicarbonate buffer solution in a concentration of 13 ng/*μ*l correspondingly just before use. The MALDI-TOF MS analysis of the resulting peptide fragments was performed using an Ultraflex II TOF/TOF mass spectrometer (Bruker Daltonics, Germany) with a reflector positive ion mode. Tryptic fragments in solution (1 *μ*l) were transferred onto the MTP 384 target plate polished steel TF mass spectrometric target and mixed with a 1 *μ*l of a matrix solution consisting of 2,4-dihydroxybenzoic acid (201346, Bruker Daltonics) in concentrations of 20 mg/ml, 50% acetonitrile in water, and 0.1% TFA. Results of 4000 laser impulses (200 impulses from 20 different points of one spot) were summed up for every spectrum. The MS data were processed using Bruker Daltonics Flex Analysis 2.4 software, and the accuracy of mass determination of peptides was fixed to 100 ppm. The correlation of the MS data with the protein sequence was done using Bruker Daltonics BioTools 3.0 software.

### 2.7. HCV Replicons

HCV subgenomic replicons were obtained by *in vitro* transcription of the RNAs from pSGR-JFH1 plasmids encoding the wild-type or mutated NS5B protein and its delivery into Huh7.5 cells by electroporation according to the standard protocol [37] and selection of the G418-resistant colonies. The cells were harvested at passages 3, 5, and 8.

### 2.8. Reverse Transcription and Real-Time PCR

HCV RNA levels were quantified by reverse transcription and real-time PCR and normalized to levels of mRNA encoding glucuronidase B (GUS). Briefly, total RNA was purified with a TRIzol reagent and cDNA was synthesized with random hexamer primer and RevertAid reverse transcriptase (Thermo Fisher Scientific, Rockford, IL, USA) according to the manufacturer's specifications. PCR was performed using primers for HCV (5′-GTCTAGCCATGGCGTTAGTA-3′ and 5′-CTCCCGGGGCACTCGCAAGC-3′) and GUS (5′-CGTGGTTGGAGAGCTCATTTGGAA-3′ and 5′-ATTCCCCAGCACTCTCGTCGGT-3′). A standard reaction mixture (13 *μ*l) contained the respective primers, cDNA equivalent to 50 ng total RNA, and qPCRmix-HS SYBR (Evrogen). The real-time PCR thermal conditions were 55°C for 5 min, 95°C for 10 min, followed by 40 cycles each at 95°C for 10 s, and 57°C for 1 min (signal collection temperature). The results were analyzed by the *ΔΔ*Ct approach.

### 2.9. Alignment of NS5B Sequences

For alignment, the following NS5B protein sequences (591 amino acid sequences of C-terminal part of the HCV polyprotein) of the corresponding HCV isolates (genotypes) were selected: 1a (H77, AAB66324), 1a (TN, ABR25251), 1a (DH6), 1a (HCV1, AAA45676), 1b (Con1, CAB46677), 1b (J4, AAC15725), 1b (DH1, KY427256), 2a (JFH1, BAB32872), 2a (JFH2, BAM28690), 2a (J6, BAA00792), 2b (J8, AFJ14788), 3a (S52, ADF97232), 4a (ED43, ADF97234), 5a (SA13, AAC61696), 6a (EUHK2, CAA72801), and 7a (QC69 accession ABN05226). Alignment was performed using Clustal Omega online service [38] at https://www.ebi.ac.uk/Tools/msa/clustalo/ site.

### 2.10. Statistical Analysis

Statistical analysis was performed using GraphPad software (GraphPad Software, San Diego, CA, USA). The results are presented as the means ± SD. Significant differences between two groups were determined using the Mann-Whitney test, whereas multiple comparisons were accessed by the Kruskal-Wallis method with the Benjamini and Hochberg procedure. Statistical differences between the activities of the mutated NS5B proteins in the absence and presence of DTT were analyzed by multiple paired Student's *t*-test.

## 3. Results

### 3.1. RNA-Dependent RNA Polymerase Activity of NS5B Protein Is Redox-Dependent

**Figure 1:**
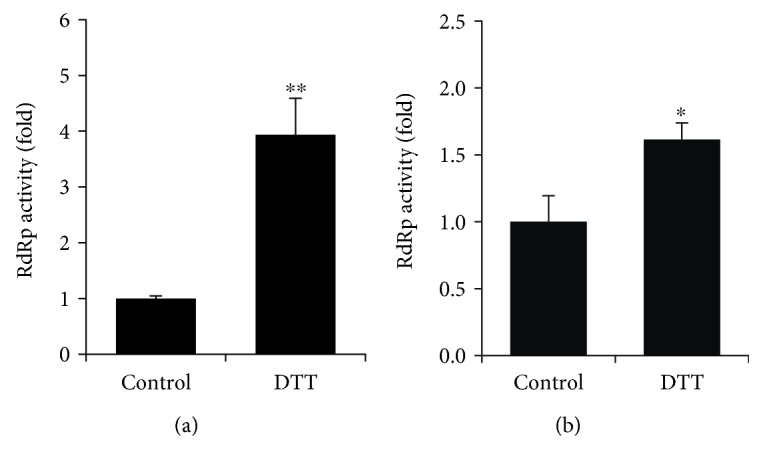
Primer-dependent (a) and *de novo* (b) RdRp activity of the recombinant NS5BΔ21 protein is stimulated in the presence of dithiothreitol. The results are presented as the mean ± SD. The control represents the untreated recombinant protein. ^∗^*p* < 0.05 and ^∗∗^*p* < 0.001 (Mann-Whitney test).

Purification of various DNA and RNA polymerases and measurement of their enzymatic activity are often performed in the presence of reducing agents such as 2-mercaptoethanol or dithiothreitol. However, to our knowledge, the reasons for the addition of these reagents are unknown. We asked therefore whether the protein's function is dependent on the presence of reducing agents. So, first, we investigated whether the RdRp activity of NS5B protein changes upon the addition of reducing agents. As the most widely used cell model for HCV replication is based on the virus genome of the JFH1 isolate of 2a genotype (gt), we expressed NS5B of 2a gt in *E.coli* and purified the recombinant protein using the vector previously developed for the expression of the polymerase of Con1 isolate [32]. It should be noted that NS5B was expressed as a truncated form lacking the C-terminal membrane-spanning motif comprising 21 amino acid residues (NS5B*Δ*21). In order to preserve the state of cysteine residues, purification was performed in the absence of reducing agents (i.e., DTT or 2-mercaptoethanol) that are routinely used in the field. The measurement of RdRp activity was performed in two assays: an efficient primer-dependent assay based on a polyA-oligoU primer-template complex and a primer-independent (*i.e.*, *de novo*) assay in which the template was presented by 3′UTR of an antigenomic RNA strand [39]. In both cases, the activity was measured by incorporation of [*α*-^32^P]UTP into RNA. The results are presented in Figure 1. It can be seen that in assays, the addition of DTT caused a significant increase in the RdRp activity of the NS5B*Δ*21 protein. In addition, the treatment of the protein with hydrogen peroxide led to a decrease in its enzymatic activity ([Supplementary-material supplementary-material-1]). This indicates that the NS5B protein acts as a redox switch.

### 3.2. NS5B Is Subject to S-Glutathionylation That Reduces RdRp Activity of the Recombinant Protein

**Figure 2:**
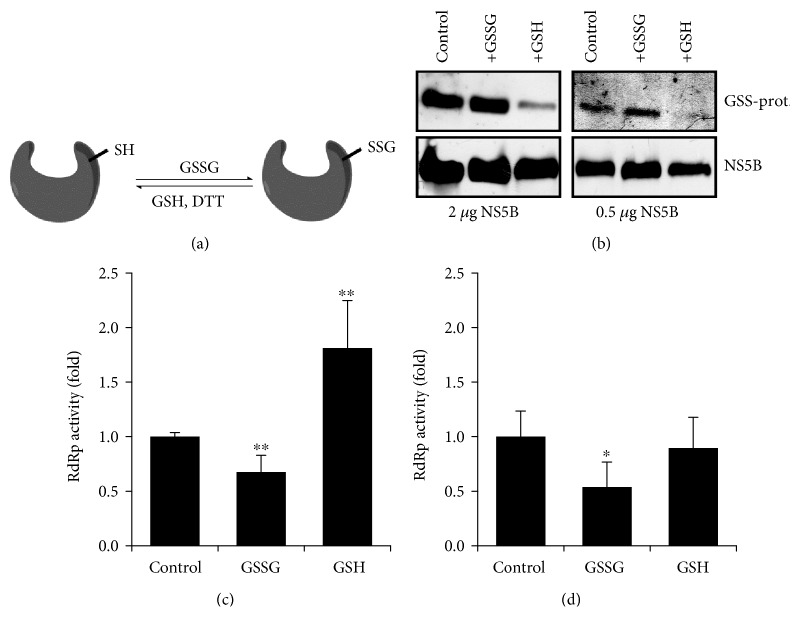
The recombinant NS5B*Δ*21 protein is subjected to S-glutathionylation which leads to reduction of RdRp activity. (a) A scheme showing impact of oxidized (GSSG) and reduced (GSH) glutathione on protein S-glutathionylation. (b) A western blot showing S-glutathionylation of the NS5BΔ21 protein. (c, d) RdRp activity of the NS5BΔ21 protein treated with glutathione isoforms or DTT, measured in primer-dependent (c) and primer-independent (d) assays. The results are presented as the mean ± SD. The control represents the untreated recombinant protein. ^∗^*p* < 0.05 and ^∗∗^*p* < 0.01 (Mann-Whitney test).

S-Glutathionylation is one of the cysteine modifications that can be removed by reducing agents. To investigate whether the NS5B protein is subjected to S-glutathionylation, the recombinant NS5B*Δ*21 protein was treated with the oxidized glutathione (GSSG) (Figure 2(a)) with subsequent analysis by Western blotting using antibodies to glutathionylated cysteine residues (anti-PSSG). As additional controls, NS5B*Δ*21 was treated with DTT or GSH. Strikingly, the western blotting revealed that the untreated recombinant NS5B*Δ*21 protein contained glutathionylated cysteines, and treatment with GSSG further increased the degree of this posttranslational modification (Figure 2(b)). Treatment with a reduced glutathione or DTT, in contrast, removed glutathione residues (Figure 2(b)), presumably by dithiol-disulfide exchange (Figure 2(a)).

Treatment of the NS5BΔ21 protein with GSSG reduced the levels of its activity in primer-dependent and primer-independent assays, whereas GSH stimulated it (Figures (2(c)) and 2(d)). Noteworthy, however, the effect of GSH was less pronounced than in the case of DTT treatment, as shown above (Figure 1). But the lower activity of reduced glutathione is in line with its lower reducing potential (*E*′ = ‐264 mV at pH 7.4), compared to that of DTT (*E*′ = ‐360 mV) [40]. Thus, the HCV NS5B protein is subject to S-glutathionylation, which modulates protein RdRp activity.

### 3.3. Identification of Cysteine Residues That Are Subjected to S-Glutathionylation

The next step was the identification of the glutathionylated amino acid residues. For that purpose, a widely used in-gel digestion procedure with trypsin was performed with subsequent analysis of the resulting peptides by MALDI mass spectrometry analysis. Two types of samples were analyzed: the recombinant freshly purified NS5B*Δ*21 protein and the same protein treated with oxidized glutathione. In both cases, the detected peptides covered 75-85% of the total protein and most of the cysteine residues. Among the cysteine residues that escaped detection in a few experiments were C14 and C39 (one experiment) and C366 (one experiment). But it should be noted that in other repeat experiments the corresponding peptides were detected thus allowing to draw conclusions about their possible modification.

The results of analysis are summarized in Table 1. As one can see, within the untreated protein, up to four cysteine residues were subjected to S-glutathionylation. Two of them were identified unambiguously: C89 and C146. S-Glutathionylation of cysteines at other positions varied between the samples. In one sample, glutathionylation was seen for the residues 170 and 274. In the other, no S-glutathionylation of C170 was detected. In contrast, two glutathione residues were found in a peptide spanning from aa 271-298 that displayed three cysteine residues at positions 274, 279, and 295. The difference in the glutathionylation status of Cys170 and cysteines of a 271-298 amino acid region might be explained by a lower level of S-glutathionylation of the respective residues in one of these samples, probably lower than the detection limit.

In the protein preincubated with GSSG, seven glutathione residues were found in the peptide digest (Table 1). As in the previous case, three of them were at cysteines 89, 146, and 274. In one out of two samples, glutathionylation was also detected for C521. Again, in both samples, the peptide aa 271-298 with two glutathione residues was seen. Taken into account that one of them should be at C274, the second one should be either at C279 or at C295. Finally, in one of the samples, the peptide aa 223-259 (bearing C223 and C243) with one glutathione residue was detected accompanied by the nonglutathionylated peptide aa 235-250. The latter indicates that another site of S-glutathionylation is presented by C223. Indeed, the second sample contained the glutathionylated peptide aa 212-231 which supported the localization of this site. It is also worth noting that in all samples, oxidation of thiols of C89, C521, and C223 and one of the pair C279/C295 into sulfenic and sulfinic acids was detected. Thus, these results indicated that the NS5B protein is glutathionylated at cysteine residues 89, 146, 170, 223, 274, 279, 295, and 521.

To find out which of these cysteine residues are conserved among HCV isolates, multiple alignment of NS5B protein variants derived from genotypes 1-7 was performed. For genotypes 1a, 1b, and 2a, three strains each were included. The alignment is shown in Supplementary [Supplementary-material supplementary-material-1], whereas Table 2 represents a summary for cysteine residues. First, the NS5B proteins from the different genotypes contained different quantities of cysteine residues: 14 for genotype 2 and 7 isolates to 21-23 for genotype 1 isolate. The residues 170, 223, 243, 279, 295, 366, and 521 were conserved among all genotypes, and cysteines 14, 89, 274, and 316 were also highly conserved: they are present in the NS5B protein of all genotypes except for one of the analyzed isolates. On the other side, cysteines 39, 146, and 303 were absent in at least two genotypes. It should also be worth noting that several genotypes were bearing cysteines in other positions that were absent in case of JFH1 and other genotype 2 polymerases ([Supplementary-material supplementary-material-1]). So, among eight cysteine residues of the NS5B protein that are subjected to S-glutathionylation NS5B, five were conservative, and two others were highly conserved among all genotypes.

### 3.4. Substitution of Most Glutathionylated Cysteine Residues in NS5B*Δ*21 Protein Leads to Reduced Enzymatic Activity of the Recombinant Protein

**Figure 3:**
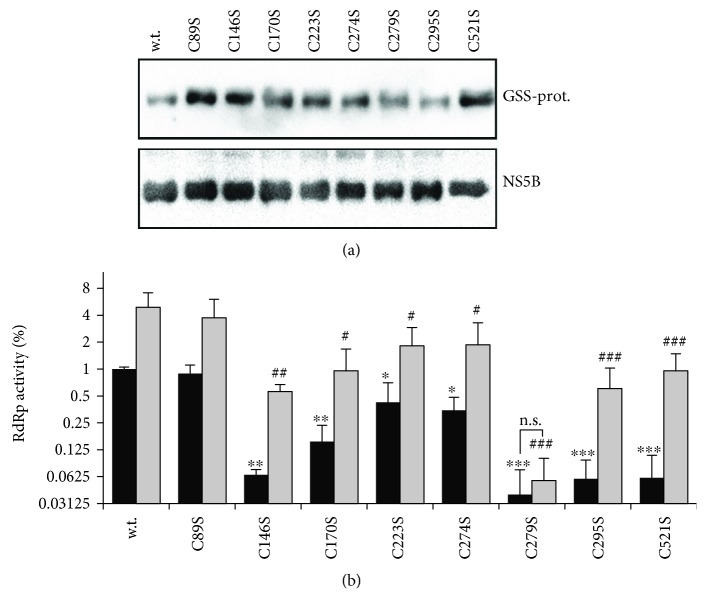
Most cysteine-to-serine substitutions in NS5B*Δ*21 decrease its enzymatic activity but do not affect its ability to be activated by a reducing agent. (a) Western blot for anti-PSSG. (b) Primer-dependent RNA polymerase activity of NS5BΔ21 mutant forms in the absence and presence of a reducing agent. The results are presented as the mean ± SD. Statistically significant differences between the activities of the mutated NS5B proteins and the wild-type enzyme were analyzed using the Kruskal-Wallis method with the Benjamini and Hochberg procedure. Influence of DTT on activity of each NS5B form was analyzed using multiple *t*-tests.

To analyze the role of glutathionylation of the identified cysteines in maintaining the enzymatic activity of the viral polymerase, a panel of NS5BΔ21 with single C→S substitution was constructed. The rationale for such mutations was to exchange the sulfhydryl group (-SH) with a hydroxyl group (-OH) that cannot be coupled with GSH or be oxidized. The expression and purification of the protein forms were carried out according to the same protocol used for the wild-type enzyme. The degree of their S-glutathionylation was analyzed using the same anti-PSSG antibodies as described above. As can be seen from Figure 3(a), not a single substitution prevented the NS5B protein from coupling with glutathione. Moreover, several mutant forms exhibited a somewhat enhanced degree of glutathionylation.

RdRp activity was then measured in a primer-dependent assay in which the same amount of the protein was present in the reaction mixture. Using the wild-type protein, no differences were observed in the *de novo* system, but the overall higher levels of nucleotide incorporation were obtained, thus ensuring better reproducibility and lower level of error. The substitution of residues 89 and 223 did not affect RdRp activity, and the change of C174 for serine only moderately reduced activity (Figure 3(b)). In contrast, other substitutions decreased the activity by >10-fold. The greatest reduction was observed for the C279S mutant, for which the incorporation of a radioactive nucleotide into RNA was close to the background. Moreover, this substitution also blocked the increase of RdRp activity upon treatment with the reducing agent. These results show that glutathionylation of several NS5B cysteines inhibits the polymerase activity of the protein.

### 3.5. Substitution of Most Glutathionylated Cysteine Residues in NS5B Protein in the Context of the Viral Subgenomic Replicon Enhanced HCV Replication

**Figure 4:**
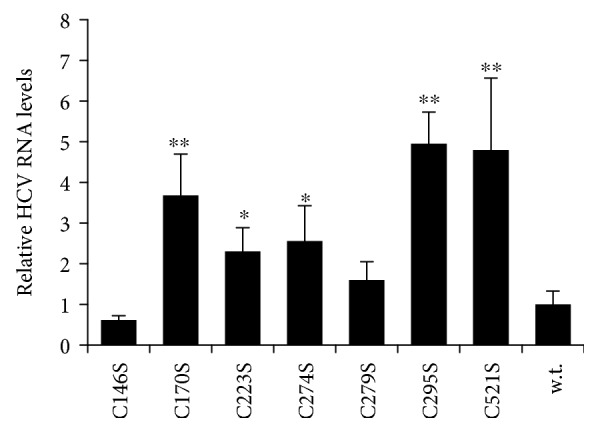
Most cysteine-to-serine substitutions in NS5B upregulate the replication of the subgenomic HCV subgenomic replicon. The results are presented as the mean ± SD. Statistical differences in replication rates between mutation-bearing and wild-type replicons were accessed by the Kruskal-Wallis method with the Benjamini and Hochberg procedure. ^∗^*p* < 0.05; ^∗∗^*p* < 0.001.

In order to verify the role of NS5B cysteine residues that are glutathionylated, on polymerase activity in the context of a viral proteome, similar mutations were introduced into a subgenomic JFH1 replicon. All the corresponding RNAs produced by in vitro transcription were electroporated into Huh7.5 cells and grown in the presence of the selection agent G418. As a negative control, the replication-incompetent variant displaying a substitution of the catalytic Asp318 with asparagine was electroporated. The results are presented in Figure 4. First, in neither out of four independent experiments, cells with an NS5B C89S substitution could be selected. This may indicate that this residue in indispensable for maintaining replication in liver cells. Substitution of C146 did not affect HCV replication efficacy. Substitutions of C179, 295, or 512 with serine residues significantly enhanced the replication of the viral genome. A less pronounced stimulation was observed for C223 and C274 substitutions. Finally, substitutions of C146 or C279 had no notable effect on HCV replication.

## 4. Discussion

Numerous reports show that hepatitis C virus alters the redox state of infected cells (summarized in [23] and other reviews) with focus on the identification of HCV-induced ROS sources and levels, identification of ROS-scavenging enzymes, and search for redox-sensitive pathways that may be implicated in virus pathogenesis. In contrast, no attempts have been made so far to analyze the impact of ROS on viral proteins in terms of redox-sensitive or redox-dependent posttranslational modifications and how these ROS-induced posttranslational modifications may change structural or enzymatic properties of viral proteins and affect virus replication as well as the development of the associated liver diseases. It is widely accepted that effects by which ROS and reactive nitrogen species deregulate any particular process are based on their ability to induce redox-sensitive posttranslational modifications. Many of those are presented by covalent modifications or cysteine thiol groups: oxidation to sulfenic (-SOH), sulfinic (-SO_2_H), or even sulfonic (-SO_3_H) acids [41]; S-glutathionylation (a formation of a mixed disulfide with glutathione) [42]; nitrosation (commonly referred to as nitrosylation—a conversion of the -SH group into the -SNO group) [43]; and many others. Numerous papers show that these posttranslational modifications play crucial roles in the regulation of many cellular enzymes and other proteins [17, 41, 43]. Among these, S-glutathionylation has a prominent role in the regulation of many pathophysiological events [42]. During last year, it became clear that this cysteine modification is implicated in the development of lung fibrosis [44], asthma [45], neural disorders such as Friedrich's ataxia [46], and other pathologies, whereas targeted regulation of S-glutathionylation in cells and tissues presents a novel strategy to treat at least some of these diseases [44]. In contrast, there are almost no data on existence of these modifications in case of proteins of HCV or other infections.

We show here that the hepatitis C virus NS5B protein that represents an RNA-dependent RNA polymerase undergoes S-glutathionylation. This finding expands the small list of viral proteins that are subject to this redox-sensitive posttranslational modification. Besides NS5B, S-glutathionylation was described for the proteases of human immunodeficiency and human T-lymphotropic viruses [18–20], the nsP2 protein of chikungunya virus [21], and NS5 protein of Zika and dengue [22]. Zika and dengue viruses belong to the *Flaviviridae*family, though to a different genus. Chikungunya virus is a member of the *Togaviridae* family that is comprised of small enveloped viruses whose genome is presented by a single-stranded nonfragmented (+) strand RNA. The NS5 protein of Zika and dengue viruses that is subject to S-glutathionylation exhibits two types of enzymatic activities: guanylyltransferase, RNA guanine-N^7^-methyltransferase, nucleoside 2′-O-methyltransferase, and RdRp [22]. S-Glutathionylation moderately decreases at least two of them: guanylyltransferase and RdRp. The nsP2 protein of Chikungunya virus is a protease, and its S-glutathionylation suppresses this activity [21]. Similarly, S-glutathionylation of HIV protease inhibits its enzymatic activity by preventing its dimerization [18, 19]. In our case, this posttranslational modification also decreased the enzymatic activity of the NS5B protein of HCV; however, the underlying mechanisms remain unknown.

S-Glutathionylation of NS5B was shown for the recombinant protein only. Unfortunately, we could not verify S-glutathionylation of the HCV NS5B protein expressed in liver cells due to technical restrictions. We tried to immunoprecipitate the protein with either NS5B or PSSG antibodies or treatment of the HCV-infected Huh7.5 cells with a biotinylated glutathione (Bio-GEE). All these approaches did not show the presence of the NS5B in the precipitates, most probably due to very weak affinity of the antibodies to this protein. Two commercially available antibodies and one in-house produced polyclonal antibody that were used for the detection of NS5B had a detection limit of 30 ng per lane of a gel. So, future endeavors should require introduction of any high affinity tag to the NS5B protein which cannot be done in the context of HCV infectious or noninfectious replication systems.

Based on existing crystal structures, it could be speculated that glutathionylation of several cysteine residues may block RdRp activity by introducing a bulky tripeptide moiety close to sites that are critical for catalysis. The first example is C223 that is located in a palm subdomain in a proximity to aspartate residue D220, which coordinates one of the magnesium ions, D225 which forms a hydrogen bond with a 2′-hydroxyl of an incoming triphosphate, or R222 that also interacts with NTP [47]. The second example is C89 that is close to the residues that form a tunnel for the RNA primer [48]. Surprisingly, the mutants with cysteines 89 or 223 substituted to serine residues exhibited enzymatic activity similar to that of the wild-type protein. In contrast, the RdRp activity of the C170S and C274S mutants was only moderately reduced. The latter is in contrast to a study of Murakami's group that demonstrated a significant reduction in the enzymatic activity of C274A mutant polymerase [49, 50]. However, such discrepancy could be explained by a different isolate of virus (JK-1 isolate of a 1b genotype compared to JFH1 isolate of 2a gt used in the present study) or by the difference in amino acid residue to which the cysteine was substituted. Next, C146S, C279S, C195S, and C521S mutant exhibited significantly reduced RdRp activity compared to the wild-type enzyme. However, the activities of all of them except C179S were upregulated by pretreatment with DTT. Again, the result for C279 is in contradiction to the papers of Murakami's group [49, 50], where C279A substitution did not affect the RdRp activity of the NS5B protein. But again, differences in viral isolate or substitution may account for this discrepancy. Nevertheless, these results indicate that several cysteine residues are important for maintaining enzymatic activity of the polymerase.

To verify that the substitutions of the cysteine residues in NS5B proteins lead to the abovementioned changes in its enzymatic activity, respective substitutions were introduced into a subgenomic HCV replicon. Stable cells lines were selected and obtained and used to measure HCV RNA levels. Strikingly, this system led to absolutely different results: a C89S mutation that had no effect on RdRp activity in vitro did not support HCV replication, whereas a series of mutations that did diminish the activity of the recombinant enzyme up-regulated HCV replication in cells. The most pronounced difference was seen in the case of the C279S mutant. This suggests the existence of at least two factors that influence RdRp activity. First, S-glutathionylation or other cysteine modifications inhibit the polymerase in cells. Second, the recombinant NS5B protein has a preference for cysteine compared to serine in the respective positions. However, it cannot be excluded that these cysteine residues can undergo other redox-sensitive modifications such as sulfenation or even sulfination that also decrease RdRp activity. It is accepted that oxidation of thiol groups into sulfenic groups may precede S-glutathionylation either through reaction with GSH or through an enzyme-catalyzed process [51]. There are several examples that both oxidation and S-glutathionylation occur for cysteine residues with reduced pKa. Mass spectrometric analysis in this study revealed both oxidized forms for several cysteines that were subjected to S-glutathionylation. Though we cannot rule out a possibility of spontaneous oxidation of these residues by air oxygen, it is tempting to speculate that at least deep oxidation (i.e., into sulfinic acids) seen for C89, C521, C223, and a pair C179/C295 could reflect a high potential of these residues to redox-sensitive posttranslational modifications. So, future studies of a wide range of cysteine modifications of the NS5B protein are required.

Both S-glutathionylation and sulfenation of cysteines rely upon hydrogen peroxide [41]. HCV infection is known to trigger enhanced production of ROS and to increase cytoplasmic H_2_O_2_ levels in particular [23, 25]. Long-lasting HCV infection also causes a significant increase in oxidized glutathione levels [31]. These facts assume that HCV infection should favor protein S-glutathionylation. As glutathionylation of the NS5B protein inhibits its enzymatic activity, one might expect a negative effect of hydrogen peroxide on virus replication. Indeed, Choi et al. reported inhibition of HCV replication by nontoxic doses of H_2_O_2_.

## 5. Conclusions

To sum up, we have shown that the NS5B protein of hepatitis C virus is subjected to S-glutathionylation and that this modification alters enzymatic activity. At the same time, results from the cell system suggest the existence of other modifications of NS5B cysteine residues that also can affect RdRp activity of the protein. So, these data point out to the necessity of future investigations of redox-sensitive posttranslational modifications of the HCV NS5B proteins as well as of other virus proteins and their role in HCV replication.

## Figures and Tables

**Table 1:** Cysteine residues of the recombinant NS5BΔ21 protein that are subjected to S-glutathionylation.

Protein		Cysteine residue													
		14	39	89	146	170	223	243	274	279	295	303	316	366	521
The recombinant NS5BΔ21	Exp1	−	−	+	+	+	−	−	+	−	−	−	−	−	−
The recombinant NS5BΔ21	Exp2	−	−	+	+	−	−	−	Two^1^			−	−	−	−
NS5BΔ21, treated with GSSG	Exp1	−	−	+	+	−	+^2^	−	Two^3^			−	−	−	+
NS5BΔ21, treated with GSSG	Exp2	−	−	+	+	−	+^2^	−	Two^3^			−	−	−	+

^1^ ^2^ ^3^The peptide comprising aa 271-298 with three cysteines (C274, C279, and C295) contained two glutathione residues. In the same analysis, no glutathionylation was revealed in a peptide of aa 271-280. A peptide aa 223-259 with two cysteines (C223 and C243) contained one glutathione residue. In a similar sample, the peptide of aa 235-250 was not glutathionylated. The peptide comprising aa 271-298 contained two glutathione residues. In the same analysis, a glutathionylated peptide of aa 251-278 is implying that one of the residues is at C274, and the second is either at C279 or C295.

**Table 2:** Variability of amino acid residues of the NS5B protein from HCV genotypes 1-7 at positions, in which the protein of JFH-1 isolate contains cysteine residues.

Gt	Isolate	14	39	**89**	**146**	**170**	**223**	243	**274**	**279**	**295**	303	316	366	**521**
1a	H77	C	S	**C**	**C**	**C**	**C**	C	**C**	**C**	**C**	C	C	C	**C**
	TN	C	S	**C**	**C**	**C**	**C**	C	**C**	**C**	**C**	C	C	C	**C**
	HCV1	C	S	**C**	**C**	**C**	**C**	C	**C**	**C**	**C**	C	C	C	**C**

1b	Con1	C	A	**C**	**C**	**C**	**C**	C	**C**	**C**	**C**	C	C	C	**C**
	J4	C	A	**C**	**C**	**C**	**C**	C	**C**	**C**	**C**	C	N	C	**C**
	DH1	C	A	**C**	**C**	**C**	**C**	C	**C**	**C**	**C**	C	C	C	**C**

2a	JFH1	C	C	**C**	**C**	**C**	**C**	C	**C**	**C**	**C**	C	C	C	**C**
	JFH2	C	C	**C**	**C**	**C**	**C**	C	**C**	**C**	**C**	C	C	C	**C**
	J6	C	C	**C**	**C**	**C**	**C**	C	**C**	**C**	**C**	C	C	C	**C**

2b	J8	C	S	**C**	**C**	**C**	**C**	C	**C**	**C**	**C**	C	C	C	**C**

3a	S52	C	S	**C**	**C**	**C**	**C**	C	**C**	**C**	**C**	A	C	C	**C**

4a	ED43	C	A	**C**	A	**C**	**C**	C	**C**	**C**	**C**	I	C	C	**C**

5a	SA13	C	S	**C**	A	**C**	**C**	C	**C**	**C**	**C**	C	C	C	**C**

6a	EUHK2	C	S	**C**	**C**	**C**	**C**	C	**C**	**C**	**C**	C	C	C	**C**

7a	QC69	S	S	A	**C**	**C**	**C**	C	V	**C**	**C**	A	C	C	**C**

## Data Availability

No data were used to support this study.

## Supplementary Materials

Supplementary Materials Figure S1: primer-dependent RdRp activity of the recombinant NS5B*Δ*21 protein is inhibited by treatment with hydrogen peroxide. Control represents the untreated recombinant protein. Figure S2: alignment of the NS5B protein derived from the virus of genotypes 1-7 and conservativeness of cysteine residues. Table S1: structure of oligonucleotides used for site-directed mutagenesis in the plasmid pET-2c-NS5B*Δ*21. Table S2: structure of oligonucleotides used for site-directed mutagenesis in the plasmid pSGR-JFH1.
