# Supplementary material for: Hepatitis C Virus RNA-Dependent RNA Polymerase Is Regulated by Cysteine S-Glutathionylation
Source: Oxid Med Cell Longev. 2019 Sep 3;2019:3196140. doi: 10.1155/2019/3196140 (PMC6800943; doi:10.1155/2019/3196140)
Supplement: Supplementary Materials — Figure S1: primer-dependent RdRp activity of the recombinant NS5BΔ21 protein is inhibited by treatment with hydrogen peroxide. Control represents the untreated recombinant protein. Figure S2: alignment of the NS5B protein derived from the virus of genotypes 1-7 and conservativeness of cysteine residues. Table S1: structure of oligonucleotides used for site-directed mutagenesis in the plasmid pET-2c-NS5BΔ21. Table S2: structure of oligonucleotides used for site-directed mutagenesis in the plasmid pSGR-JFH1. [file 3196140.f1.docx]

**Supplementary figures**

**
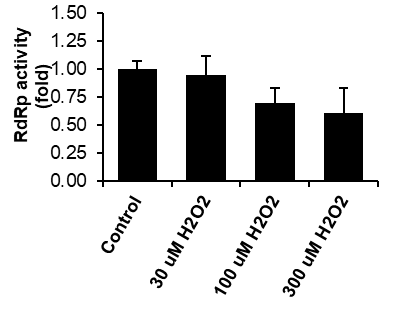
**

FIGURE S1. Primer-dependent RdRp activity of the recombinant NS5BΔ21 protein is inhibited by treatment with hydrogen peroxide. Control represents the untreated recombinant protein.


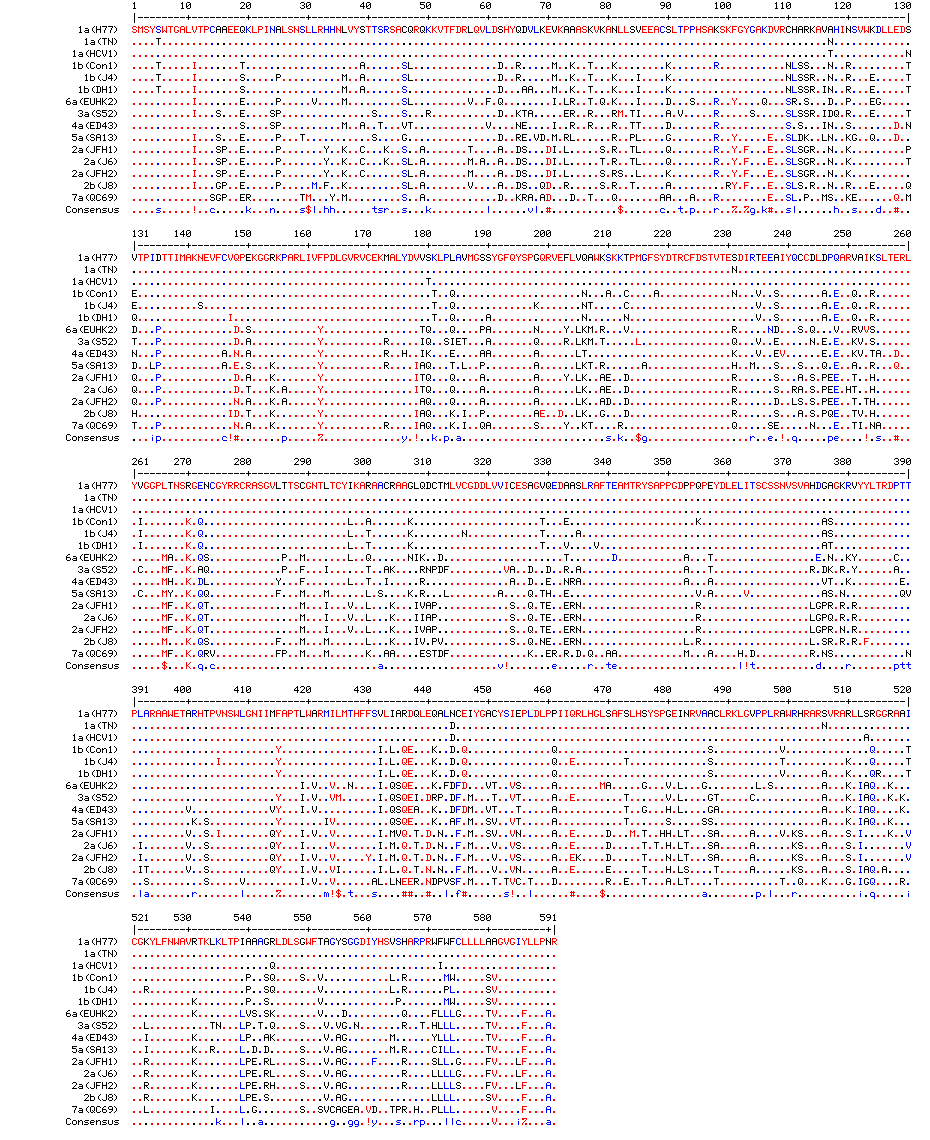


FIGURE S2. Alignment of NS5B protein derived from the virus of genotypes 1-7 and conservativeness of cysteine residues.

Table S1.

Structure of oligonucleotides used for site-directed mutagenesis in a plasmid pET-2c-NS5BΔ21

| Mutation | Fragment | Orientation | Site | Sequence (5'→3') |
| --- | --- | --- | --- | --- |
| 89, 146, 170 | I | sense | EcoRI | TAATACGACTCACTATAGGG |
| 89 | I | antisense | - | TGGAGTCAACTGGCTCGCCTCCT |
| 89 | II | sense | - | AGGAGGCGAGCCAGTTGACTCCA |
| 146 | I | antisense | - | ATGAGGTGTTCTCCGTGGAC |
| 146 | II | sense | - | TGAGGTGTTCAGCGTGGAC |
| 89, 146 | II | antisense | HindIII | GATGAAGCTTTTGTGTAATGTCATAGAGGGCCATTTTCTCGCAGACC |
| 170 | II | antisense | HindIII | GATGAAGCTTTTGTGTAATGTCATAGAGGGCCATTTTCTCGGAGACC |
| 223 | I | sense | NcoI | AGAACCATGGGTTTTTCGTATGATACCCGAAGCTTCG |
| 274, 279, 295, 521 | I | sense | NcoI | AGAACCATGGGTTTTTCGTATGATACCCGATGCTTCG |
| 274 | I | antisense | - | TCTGTAACCGCTGGTTTGACC |
| 274 | II | sense | - | GGTCAAACCAGCGGTTACAGA |
| 279 | I | antisense | - | GCTGGCGCGAGAACGTCTGTAAC |
| 279 | II | sense | - | GTTACAGACGTTCTCGCGCCAGC |
| 295 | I | antisense | - | CACATAGCTTGTGATGGTGTTACC |
| 295 | II | sense | - | GGTAACACCATCACAAGCTATGTG |
| 521 | I | antisense | - | GAGATATCGGCCGCTAACTGCCGCTT |
| 521 | II | sense | - | AAGCGGCAGTTAGCGGCCGATATCTC |
| 223, 274, 279, 295, 521 | II | antisense | XhoI | GCTAGTTATTGCTCAGCGG |

Table S2.

Structure of oligonucleotides used for site-directed mutagenesis in a plasmid pSGR-JFH1

| Mutation | Fragment | Orientation | Site | Sequence |
| --- | --- | --- | --- | --- |
| C89S, C146S, C170S, C223S | I | sense | BsrGI | GTTGCCAATCAACCCTTTGAGT |
| C89S | I | antisense | - | GGAGTCAACTGGGACGCCTC |
| C89S | II | sense | - | GAGGCGTCCCAGTTGACTCC |
| C146S | I | antisense | - | GTCCACGGAGAACACCTCAT |
| C146S | II | sense | - | ATGAGGTGTTCTCCGTGGAC |
| C170S | I | antisense | - | GAGGGCCATTTTCTCGGAGACC |
| C170S | II | sense | - | GGTCTCCGAGAAAATGGCCCTC |
| C223S | I | antisense | - | GAGGGCCATTTTCTCGGAGACC |
| C223S | II | sense | - | TACCCGATCCTTCGACTCAAC |
| C89S, C146S, C170S, C223S | II | antisense | SnaBI | ACGTCTGTAACCGCAGGTTTG |
| C274S, C279S, C295S, D318N, C521S | I | sense | SnaBI | GGTCTGCGAGAAAATGGCCCTC |
| C274S | I | antisense | - | CGTCTGTAACCGGAGGTTTGAC |
| C274S | II | sense | - | GTCAAACCTCCGGTTACAGACG |
| C279S | I | antisense | - | AcGTCTGTAACCGGAGGTTTG |
| C279S | II | sense | - | ACCTGCGGTTACAGACGTTCTCGAGCCAGTGGGGTGCTAAC |
| C295S | I | antisense | - | TTCACATAGGATGTGATGGTG |
| C295S | II | sense | - | CACCATCACATCCTATGTGAA |
| D318N | I | antisense | - | ACTAGGTCATTGCCGCATACCAGC |
| D318N | II | sense | - | GCTGGTATGCGGCaATGACCTAG |
| C521S | I | antisense | EcoRV | AAGAGATATCGGCCGGAAACGG |
| C274S, C279S, C295S, D318N | II | antisense | EcoRV | AAGAGATATCGGCCGCAAACGG |
